# Effectiveness and cost-effectiveness of a multimodal, physiotherapist-led, vocational intervention in people with inflammatory arthritis: study protocol of the Physiotherapy WORKs trial

**DOI:** 10.1186/s41927-023-00357-4

**Published:** 2023-09-20

**Authors:** N. F. Bakker, S. F. E. van Weely, N. Hutting, Y. F. Heerkens, J. A. Engels, J. B. Staal, M. van der Leeden, A. Boonen, W. B. van den Hout, T. P. M. Vliet Vlieland, J. Knoop

**Affiliations:** 1 Department of Orthopaedics, Rehabilitation and Physical Therapy, Leiden University Medical Center, Albinusdreef 2, P.O.Box 9600, 2300 RC Leiden, the Netherlands; 2 Research Group Occupation & Health, HAN University of Applied Sciences, Nijmegen, the Netherlands; 3 Musculoskeletal Rehabilitation Research Group, HAN University of Applied Sciences, Nijmegen, the Netherlands; 4 Radboud Institute for Health Sciences, IQ Healthcare, Radboud University Medical Centre, Nijmegen, the Netherlands; 5 Reade, Rehabilitation and Rheumatology, Amsterdam, the Netherlands; 6 Amsterdam Movement Sciences, Musculoskeletal Health, Amsterdam, The Netherlands; 7 Department of Internal Medicine, Division of Rheumatology, Maastricht University Medical Centre, Maastricht, the Netherlands; 8 Care and Public Health Research Institute (CAPHRI), Maastricht University, Maastricht, the Netherlands; 9 Department of Biomedical Data Sciences, Leiden University Medical Center, Leiden, the Netherlands

**Keywords:** Vocational rehabilitation, Rheumatoid arthritis, Axial spondyloarthritis, Randomized controlled trial, Physiotherapy

## Abstract

**Background:**

Although reduced work ability is a substantial problem among people with inflammatory arthritis (IA), work ability is an underexposed area in clinical practice. Evidence on vocational interventions in IA is limited, but favourable results of delivery by a physiotherapist (PT) warrant the need for further research. Therefore, we aim to evaluate the (cost-)effectiveness of a multimodal, PT-led, vocational intervention in (self-)employed people with IA compared to usual care.

**Methods:**

This randomized controlled trial will include 140 people with rheumatoid arthritis (RA) or axial spondyloarthritis (axSpA) who are (self-)employed and have reduced work ability (Work Ability Index – Single Item Scale (WAS) ≤ 7/10) and/or RA/axSpA related sick leave (≤ 6 months). Participants will be randomized 1:1 to the intervention or control condition (usual care). The intervention, delivered by primary care PTs, will be personalized to each patient, consisting of 10 to 21 sessions over 12 months. The intervention will be multimodal, comprising of 1) exercise therapy and a physical activity plan, 2) education/self-management support, 3) work-roadmap to guide participants in finding relevant other care, with optionally 4) online self-management course and 5) workplace examination. Assessments will be performed at baseline and after 3, 6, and 12 months. The primary outcome measure of effectiveness is work ability, as measured with the WAS at 12 months. For the cost-effectiveness analysis, the EuroQol (EQ-5D-5L), self-reported healthcare use, sick leave and productivity while at work will be used to estimate the trial based cost-utility from a societal perspective. A process evaluation, including assessments of adherence and treatment fidelity, will be undertaken using the registrations of the PTs and semi-structured interviews at 12 months follow-up in a random sample of the intervention group.

**Discussion:**

The results of this study will provide insights in the (cost-)effectiveness of a multimodal, PT-led, vocational intervention in people with IA and a reduced work ability.

**Trial registration:**

This study is registered in the International Clinical Trial Registry Platform (ICTRP) under number NL9343.

## Background

Rheumatoid Arthritis (RA) and axial SpondyloArthritis (axSpA) are chronic rheumatic diseases, characterized by inflammation of the joints, resulting in joint pain, stiffness, fatigue [1–3] and reduced health-related quality of life [3, 4]. These diseases usually begin in the fifth (RA) or third (axSpA) decade of life and thus affect people of working age [3, 5]. Despite breakthroughs in the pharmacological treatment, work ability of people with RA and axSpA is substantially reduced compared to the general population [6–8] and is characterized by substantial job loss [6], up to 38% of people with RA or axSpA lose their jobs already within the first few years of diagnosis [9], sick leave [4], and decreased productivity while at work (i.e., presenteeism) [10]. This causes considerable economic consequences for individuals as well as society [4, 11]. European yearly indirect costs in people with RA and axSpA were in 2015 estimated around €4.000 to €5.000 per person [12, 13].

Although reduced work ability is an important problem in RA and axSpA, the number of studies on vocational interventions for these patient groups is limited. Such interventions are referred to as job loss prevention, occupational rehabilitation or vocational rehabilitation and may be delivered by physiotherapists (PT), occupational therapists (OT), social workers, psychologists or other professionals, either monodisciplinary or multidisciplinary. Two recent systematic reviews [9, 14], including 6 randomized controlled trials (RCTs) [15–20] and 1 pilot-RCT [21], evaluated supervised vocational interventions on work-related outcomes in RA or axSpA. In these studies, the intervention was delivered by a multidisciplinary team [16, 18], or monodisciplinary by an OT [17, 20, 21], OT or PT [19] or rehabilitation counselor [15]. The RCTs showed conflicting evidence and overall a small effect on work-related outcomes. Interestingly, both studies [16, 18] evaluating a multidisciplinary vocational intervention on work-related outcomes found no effect, but all five studies involving monodisciplinary vocational interventions on work-related outcomes, reported an positive effect. Three studies found a medium effect [17, 20, 21] but from the results of two studies no effect sizes can be estimated [15, 19].

Although it is difficult to compare the magnitude of the treatment effects in the studies on PT or OT led vocational interventions due to variety in work-related outcome measures, the delivery of vocational interventions by a PT trained to treat people with inflammatory arthritis (IA) seems promising. Firstly from a patient-perspective but also from an economic perspective, as less expensive than a multidisciplinary intervention. In addition to the abovementioned positive results in IA, PT-led interventions have also been found to be effective on work-related outcomes in musculoskeletal pain in multiple studies [22–24]. Especially in patients with IA, delivery of vocational interventions by PTs may be an attractive option, as physiotherapy is relatively often used in this patient group (i.e., 25–50% of patients with IA visit a PT per year in the Netherlands [25]).

In summary, studies on the effectiveness of vocational interventions delivered by a PT in people with RA or axSpA and reduced work ability are scarce. The aim of the study is therefore to evaluate the effectiveness and cost-effectiveness of a PT-led vocational intervention for people with RA or axSpA and reduced work ability as compared to usual care.

## Methods

### Study design

**Fig. 1:**
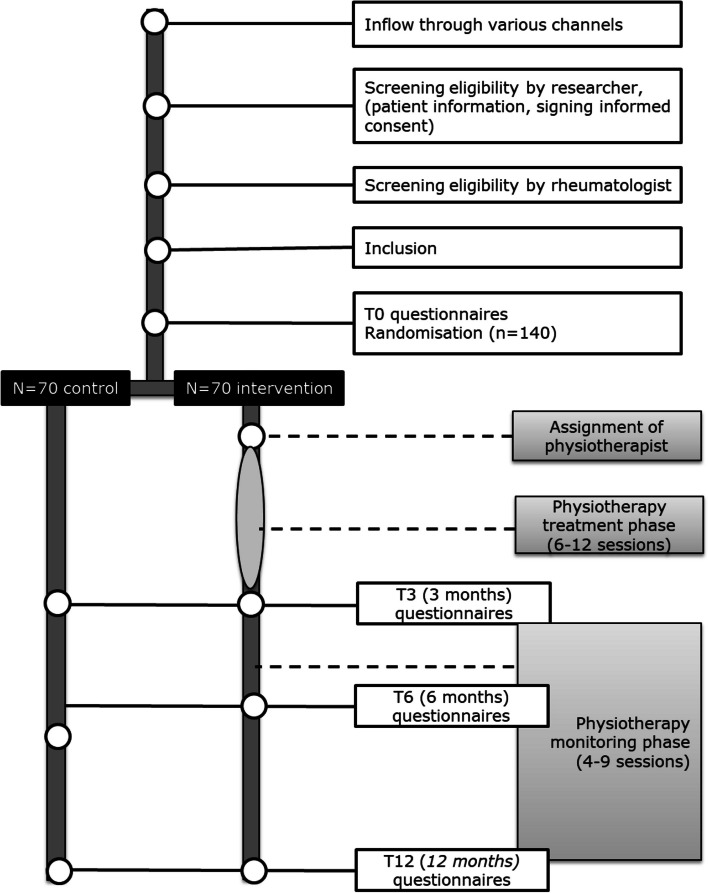
Study flowchart [26]

In a Dutch nationwide single-blind RCT, the effectiveness and cost-effectiveness of a multimodal, PT-led, vocational intervention compared to usual care in (self-)employed people with RA or axSpA and a reduced work ability will be evaluated. Measurements will take place at baseline, 3, 6 and 12 months (primary endpoint). An overview of the study is provided in the flowchart (Fig. 1).

### Study setting

The intervention will be delivered by primary care PTs. People in the Netherlands have direct access to a PT. Since 2012, the costs of physiotherapy are not reimbursed by the basic health insurance. Full or partial reimbursement can be obtained by means of a complementary health insurance, otherwise patients must pay out-of-pocket. In the Netherlands, 76% of the people with RA or axSpA have such a complementary health insurance [25]. In this study, participants paid for the physiotherapy sessions using the same approach as described above.

### Study context

In the Netherlands, employers are legally obliged to have a contract with an occupational healthcare service [27]. Employees with prolonged sick leave (i.e., ≥ 6 weeks) receive care from an occupational physician affiliated to such an occupational healthcare service [28, 29]. Employees can also approach an occupational physician in case of work-related problems as a preventive measure. Self-employed people in the Netherlands are expected to protect themselves against the risk of disability by taking out a disability insurance. In practice, however, only about a quarter of the self-employed people have a disability insurance [30]. People without a disability insurance are not entitled to counseling and benefits in the event of sick leave or job loss.

### Participants

The study population will consist of (self-)employed people with RA or axSpA and reduced work ability.

#### Inclusion criteria

1. Adult people (≥ 18 years) with a clinical diagnosis of RA or axSpA (confirmed by a rheumatologist),
2. Being (self-)employed for a minimum of 12 h/week.
3. Moderate to poor work ability (Work Ability Index-Single Item Scale (WAS) ≤ 7/10 [31] related to RA or axSpA and/or a self-reported history of sick leave in the last six months, related to RA or axSpA.
4. Self-reported limitations in physical functioning related to RA/axSpA.
5. Willingness to use physiotherapy (nearby participant).
6. Willingness to pay for physiotherapy (through (partial) complementary health insurance or out-of-pocket).
7. Sufficient command of Dutch language.

#### Exclusion criteria

1. Pensionable age within two years, because this minimizes the perceived necessity of a vocational intervention.
2. Persistent sick leave period of more than 6 months, because of presumed reduced potential of a vocational intervention with a prolonged sick leave period.
3. Comorbid disease or situation other than RA or axSpA (including elective hospital admissions or major surgery in the coming 12 months) that significantly affects work ability, as this work ability is unlikely to be affected by an intervention targeting arthritis-related work disability.
4. Pregnancy, because maternity leave during the intervention and follow-up period will hamper the execution of the intervention and interpretation of assessments.
5. Being in a formal labour dispute, because this generally indicates non-health factors dominating perceived work ability which cannot be influenced by the intervention.

### Study procedures

During the recruitment period, information about the study will be shared with all rheumatology departments and outpatient clinics in the Netherlands and through various public media. Potentially eligible participants interested in the study receive information about the study as much as necessary from a medical ethical perspective, with only minimal information about the content of the experimental intervention, namely that it is PT-delivered and includes exercise therapy and advices regarding work, and will be subsequently screened by the researcher (NB). If a potential participant meets the eligibility criteria and consents, the treating rheumatologist will be contacted to confirm the clinical diagnosis. Potential participants will be included after receiving a confirmation of the diagnosis and informed consent. Participants will formulate and rate one specific work-related limitation in physical functioning and two specific other limitations in physical limitations in daily life, using the Patient Specific Complaints Numeric Rating Scale (PSC NRS) [32] during a telephone conversation with the researcher (NB). Baseline data (T0) and follow-up assessments, administered at 3-months (T3), 6-months (T6) and 12-months (T12) after baseline, will be collected through online questionnaires.

### Randomization and blinding

Randomization will be performed by the researcher (NB) using the software Castor (Castor. EDC©) at the participant level, in blocks of varying sizes (2–4-6 participants, with block size randomization) in a 1:1 ratio. Randomization will be stratified for disease (RA vs. axSpA), disease duration (< 5 vs. ≥ 5 years since diagnosis), and current sick leave (yes vs. no). We will stratify for disease duration because in the first few years after the diagnosis pharmacological treatment has started and symptoms could possibly fluctuate more than later in the disease course. The researcher will not be blinded for treatment allocation of the participants due to logistic reasons regarding contact with participants and treating PTs, but the researchers conducting the primary analyses will be blinded for the group allocation.

After completion of the baseline assessment, the participants will be informed by the researcher (NB) about their assigned condition (intervention/control). Given the nature of the intervention, participants and PTs involved in the treatment cannot be blinded to the treatment allocation.

### Intervention

#### Recruitment and training of PTs

The intervention will be delivered by primary care PTs in the neighbourhood of the participants’ home. PTs will be primarily recruited from the ‘ReumanetNL’ network (www.reumanetnl.nl), a nationwide network of PTs with expertise in treating people with RA or axSpA. Participants in the experimental group who prefer to be treated by the PT that they are familiar with, are given this opportunity if this PT is willing to follow the study training and consent with the treatment protocol. To minimize contamination and because its (cost-)effectiveness is still unknown, PTs in the experimental arm are prohibited to provide the experimental intervention to participants outside the study and to participants in the control arm during their participation in the study.

Participating PTs are instructed to comply with the current Dutch physiotherapy guidelines for RA [33] and physiotherapeutic management recommendations for axSpA [34]. In addition they will have access to an online training environment and receive the treatment protocol on paper. The mandatory training for PTs will consist of multiple e-learnings on i) integration of work in the physiotherapy treatment, ii) Dutch occupational healthcare system and work-related laws/regulations, and iii) treatment protocol including a live question session. If deemed necessary, supplemental disease-specific trainings could be followed. The total duration of this mandatory training is approximately five hours. Participating PTs can contact a study team member with extensive expertise in physiotherapy for these populations at any time during the trial.

### Intervention

The experimental group will receive a multimodal, PT-led, vocational intervention based on an integration of existing guidelines, programs, materials and clinical knowledge and experience [9, 33–39]. This intervention was developed according to the Medical Research Council framework for developing and evaluating complex interventions [40], in co-creation with people with RA or axSpA, PTs, (occupational) healthcare professionals (occupational physician, labour expert, OT, rheumatologist, nurse specialist), and researchers during six group meetings and was tested for feasibility in four patients. This intervention development process did result in several adaptations of the (draft) intervention, such as elaboration of the work-roadmap and the development of training courses for PTs in the trial [41].

The intervention consists of work-focused modalities embedded in the conventional physiotherapy treatment and comprises 10 to 21 PT sessions of 30 min (combination of face-to-face, online or telephone-based sessions) over a 12-month period, delivered in four steps (see Table 1). Due to the complexity of work ability, a multimodal approach with a long follow-up period is considered necessary to achieve sustainable changes in work ability. This approach should enable the PT to (i) gradually increase the intensity of the exercise therapy, (ii) monitor whether the participant succeeds in reaching sustainable lifestyle changes (e.g., being more physically active), (iii) adequately signpost the participant to other professionals over time, and (iv) monitor the impact of any work-related adaptations if applicable. The intervention will be multimodal, consisting of a combination of the following three mandatory and two optional treatment modalities:

**Table 1:** Structure of the intervention

Step	Aim	Session	Content of step
1	Unravelling the participants’ work-related problems in relation to RA/axSpA	Preparation of the first face-to-face consultation with the PT	Participants provide information about their work context using parts of a previously developed questionnaire by de Buck et al. [42]. This information and the three specific (work-related) limitations in physical functioning as measured with the PSC NRS by the researchers before randomization, will be shared with the PT
		First consultation	The PT performs a systematic assessment aimed at clarifying the RA/axSpA-related work problems and problems encountered in daily life, during a semi-structured dialogue with elements of motivational interviewing using the patient specific goal setting approach (PSG) [43]. During this dialogue, participants are supported to define relevant work treatment goals aligned to their specific (work-related) limitations in physical functioning
2	Developing a personalized, multi-modal treatment plan	Second consultation	A personalized treatment plan will be formulated including treatment modalities and frequency of supervision, based on the participants’ needs and preferences in a shared decision making dialogue with the PT. PTs are offered a decision tree and form, to guide them in personalizing the intervention to the participants’ needs
3	Execution of the personalized, multi-modal plan	4 to 10 face-to-face sessions within the first 3 months	Comprising of the following treatment modalities: 1. Personalized exercise therapy including a personal physical activity plan 2. Personalized education and self-management support 3. Personalized ‘work-roadmap’ Optionally (if considered beneficial by PT and agreed by the participant): • A workplace examination • An online self-management course
4	Monitoring of the personalized, multi-modal plan	4 to 9 ‘booster’ sessions within the following 9-months (online, telephone-based or face-to-face)	To facilitate and guide adherence to the formulated treatment plan and to check the achievement of treatment goals

#### Mandatory modalities

1. Exercise therapy including personal physical activity plan;
2. Education and self-management support;
3. ‘Work-roadmap’: adequately signposting participants to other professionals with regard to optimizing their work ability.

#### Optional modalities

4. Online self-management course;
5. Workplace examination.

These modalities will all be adjusted to the individuals’ needs based on the defined work treatment goals and aligned to the individuals’ specific (work-related) limitations in physical functioning. In Table 2 an extensive description of the content of the intervention modalities is given.

**Table 2:** Content of the different treatment modalities

Treatment modality	Content
Exercise therapy including a personal physical activity plan	A personalized exercise therapy plan will be formulated, based on current physiotherapy guidelines for RA [33] and recommendations for axSpA [34] and consists of guided PT and formulating a personal physical activity plan. The guided exercise therapy will specifically target the work-related problems identified in step 1 and 2 and aims at modifiable factors for physical fitness (strength, aerobic capacity, mobility), of which improvements have been linked to improved work ability [44, 45]. If indicated by the PT and agreed upon by the participant a behavioral graded activity approach is included [46]. The personalized physical activity plan will be jointly developed by the PT and participant and aims to integrate physical activity into the participants’ daily life in a sustainable way
Education and self-management support	Personalized information (written and oral) on self-management strategies will be provided, focusing on work-related problems for people with RA/axSpA (i.e., balance between load and capacity at work, coping with fatigue/pain/energy level, discussing work disability with colleagues) and personal barriers and facilitators
‘Work-roadmap’	The work-roadmap guides the participant in when and how to get the necessary support from different (occupational) healthcare professionals. A step-by-step roadmap is used and tailored for the individual participant*,* based on the individuals’ work context and their work-related limitations in physical functioning as determined in the screening process. The PT will act as a coach to support the participant in taking actions as described in the roadmap
**Optionally (if considered beneficial by PT and agreed by the participant)**	
An online self-management course	To further optimize self-management and empowerment skills specifically focusing on work. This course is paid from the study budget and consists of two 1,5 h online sessions. The course is organized in groups of four to six participants of the intervention arm and is supervised by an experienced coach in rheumatic diseases and work-related problems (www.annemiekdecrom.nl)
A workplace examination	PT visiting the workplace or by pictures/video of the participant working at the workplace. Targeting necessary adaptations at the workplace and/or (help with preparing) a dialogue between employee and supervisor or colleagues to facilitate possible work adaptations and acceptance from the supervisor and colleagues [46]

In addition to the intervention, participants are allowed to receive usual care.

### Control

The control group will continue their usual care.

### Outcome measures and data collection

The primary outcome is the participants’ reported work ability assessed by the Work Ability Index-Single Item Scale (WAS) [31]. The secondary outcomes are divided into four categories: 1) work-related outcomes; 2) clinical outcomes; 3) healthcare use and costs from the societal perspective and 4) expectancy of the treatment and global perceived effect. A detailed description of all outcome measures is shown in Table 3 and the timepoints on which they are assessed are displayed in Table 4.

**Table 3:** Description of outcome measures

Measures	Description
**General characteristics**	
Sociodemographic and work characteristics; comorbidity	Year of birth, gender, weight and height to calculate the body mass index, civil state, household composition, education level, work status, type of work and employment, number of working hours and days a week, smoking, comorbidities
Disease characteristics (retrieved from rheumatologist)	Clinical diagnosis (RA/axSpA), year of diagnosis, prescribed medication, erythrocyte sedimentation rate (ESR, mm/hr) or level of C-reactive (CRP, mg/L) and disease activity measured by Bath Ankylosing Spondylitis Disease Index (BASDAI) [47] for axSpA and Disease Activity Score 28 joint count (DAS28) for RA [48]) will be retrieved from the participant’s rheumatologist
**Primary outcome**	
WAS (Work Ability Index-Single Item Scale) [31]	The WAS is a responsive outcome measure to assess the status and progress of work ability and is highly predictive for future sick leave. It consists of one scale (NRS) indicating the level of work ability a participant experiences at the moment of measuring ranging from 0 = completely unable to work at all, to 10 = work ability at its best, and distinguishing the following well-accepted categories: 0–5 = poor, 6–7 = moderate, 8–9 = good, and 10 = excellent work ability
**Secondary outcomes**	
**Work-related**	
WPAI (Work Productivity and Activity Impairment questionnaire) [49, 50]	The WPAI assesses presenteeism, absenteeism and productivity at work specific for RA patients [50] or axSpA patients [49]. It consists of six items from which overall score for % of overall work restriction due to RA/axSpA can be calculated
Job satisfaction [51]	This one item questionnaire, derived from Linton and Halldén 1998 [51], assesses job satisfaction on a 0–10 scale (0 = totally dissatisfied, 10 = very satisfied)
Self-efficacy at work [52]	A single question, derived and translated from the work subscale of the Basic Psychological Need Satisfaction and Frustration Scale (BPNSFS) [52], measuring self-efficacy at work on a 1–5 scale (1 = totally disagree, 5 = totally agree)
**Clinical**	
NRS pain (Numeric Rating Scale pain) [53]	Pain severity in the past 7 days on NRS; 1 item on 0–10 scale (0 = no pain, 10 = worst pain possible). Clinically relevant difference is a two point change between baseline and follow-up
NRS fatigue (Numeric Rating Scale fatigue) [34]	Fatigue in the past seven days on NRS; 1 item on 0–10 scale (0 = no fatigue, 10 = worst possible fatigue). Clinically relevant difference is a two point change between baseline and follow-up
PSC NRS (Patient specific Complaints Numeric Rating Scale) [32]	The PSC NRS is an individualized outcome measure designed to detect changes in a participants’ perception of functioning over time. It consists of three scales (NRS) indicating the level of difficulty participants encounter while executing activities that are most relevant for them ranging from 0 = easy, to 10 = impossible to do. In this study the first activity has to be work-related, the next two activities may also concern activities related to other domains of daily life
PROMIS-SF Physical Function PF-10 (Patient Reported Outcome Measurement Information System‐Short Form Physical Activity) [54–56]	PROMIS is a standardized metric for measuring health across chronic diseases, developed using the item response theory. In this study the PROMIS Short Form v2.0— Physical Function 10a will be used to measure the patient reported physical function. The questionnaire consists of 10 questions. All questions have five answer options ranging from 1 = easy to 5 = impossible to do. From the raw score a T‐score is derived, with the Dutch/Flemish population mean and a standard deviation. A high score indicates a poor patient reported physical function
^a^ BASFI (Bath Ankylosing Spondylitis Functional Index) [47, 57]	BASFI is a validated instrument to assess the degree of limitations in activities in patients with axial spondyloarthritis. It includes 10 questions on how well activities went in the past week on a NRS scale, ranging from 0 = easy to 10 = impossible to do. The BASFI score is calculated by taking the mean of the score of the 10 individual questions. Scores can range from 0 to 10, with a high score referring to severe limitations
m-SQUASH (Modified Short Questionnaire to Assess Health-enhancing physical activity) [58]	m-SQUASH is a 17 item questionnaire to assess physical activity level and includes items regarding physical activities, sports activities and work activities
HADS (Hospital Anxiety and Depression Scale) [59]	The HADS measures levels of anxiety and depression in the past four weeks. It comprises seven items on anxiety and seven items on depression ranging from 0 to 3. Scores higher than 8 on the anxiety and depression items separately indicate problems concerning anxiety and depression
**Health care use, costs and quality of life**	
Societal costs [60]	Including general practitioner visits, outpatient visits, hospital days, rehabilitation center days, nursing home days, physiotherapy use, home care, (change in) medication use, informal care, costs for patients related to RA/axSpA, work situation and productivity related costs. Similar questionnaires have been used in previous studies on physiotherapy in inflammatory arthritis
EuroQol (EQ‐5D‐5L) [61, 62]	The EuroQol is a standardized instrument including 5 dimensions of health (mobility, selfcare, daily activities, pain/complaints and anxiety/depression), resulting in a score anchored at 0–1, with a higher score indicating better health. It also includes a ‘thermometer’, a visual analog scale with a score ranging from 0 (worst possible health) to 100 (perfect health)
**Expectancy of treatment and global perceived effect**	
Expectancy of intervention	One question constructed by the research group to ask the participant to what extent they expect the intervention to effect their work ability. On a NRS scale ranging from 0 (not at all) to 10 (very)
GPE (Global Perceived Effect)	Contains the anchor question on the perceived effect: “Has the vocational PT-led intervention changed your daily functioning?”

^a^Measured only in the study population of axial spondyloarthritis patients

**Table 4:**
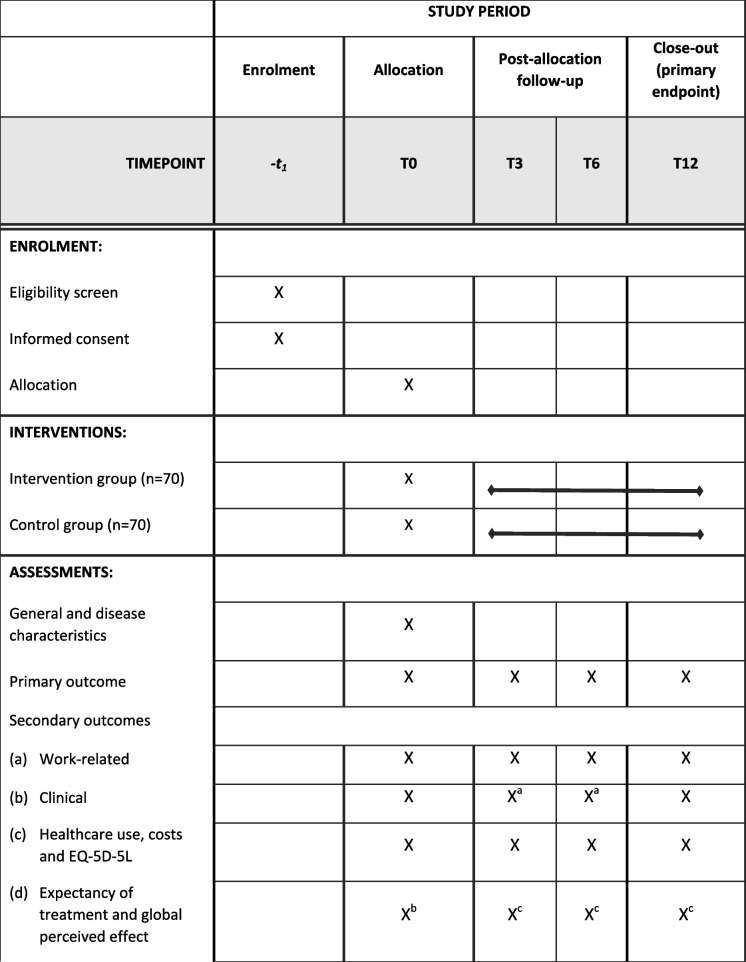
SPIRIT flowchart: Outcome measures at the different timepoints

^a^The PSC NRS will not be measured at T3 and T6

^b^The expectancy of the treatment will only be measured at T0

^c^The GPE will not be measured at T0

At baseline, disease characteristics will be retrieved from the participants’ rheumatologist. Data from patient questionnaires will be collected and stored in the online database OnlinePROMS© (2020, Interactive Studios BV, Rosmalen, the Netherlands).

#### (Serious) Adverse events

All participants and PTs will be asked to immediately and proactively report serious adverse events (SAE) or adverse events (AE) to the researchers. SAEs and AEs, directly related to the intervention, will be recorded and followed until they have abated, or until a stable situation is reached. The researchers will report all intervention-related SAEs to the sponsor without undue delay after obtaining knowledge of the events.

#### Sample size calculation

The sample size was calculated with a conservatively estimated between-group effect size of 0.5 on the primary outcome measure WAS [31], based on an expected improvement in the experimental group from 6 (moderate work ability) to 8 (good work ability), an expected improvement in the control group from 6 to 7 (to take into account a potential ‘regression to the mean effect’) and a standard deviation of 2 [18, 46]. Based on previous studies [17, 21], this between-group effect size can be considered feasible. Based on two-sided testing, a significance level of 0.05, a power of 0.8, and this expected between-group effect size, 126 participants have to be recruited. Based on trials on comparable vocational interventions in IA [15, 17, 19], we took into account a drop-out rate of 10% (i.e., 126/90 * 100), which resulted in a sample size of 140 participants (70 in each arm). Considering a threefold higher prevalence of RA over axSpA in the Netherlands, we expect both groups to comprise more people with RA than axSpA.

#### Process evaluation

The process of the trial will be evaluated both quantitatively and qualitatively, comparable to previous and ongoing other trials from our group [63, 64]. For the quantitative evaluation, PTs will report process parameters after each treatment session (in OnlinePROMS®), including participant adherence, number of treatment sessions, the content of the applied treatment, and adverse events. Based on these parameters, PT treatment fidelity can be assessed. For the qualitative evaluation, a random sample of 10 participants and 10 PTs will be selected and invited for semi-structured interviews to discuss experiences, barriers and facilitators with regard to the intervention. The semi-structured interviews will be conducted after the selected participant has completed the 12-month assessment.

### Statistical analysis

#### Primary analysis

A multilevel, regression analysis – with levels of patient and time point – will be performed using linear mixed modeling. Primary outcome measure WAS will be analyzed as a dependent variable, using the study group (intervention vs control), stratification variables (i.e., disease (RA vs. axSpA), disease duration (< 5 vs. > 5 years), current sick leave at randomization (yes or no)) and other potential confounders as independent variables. All analyses will be performed according to the intention-to-treat principle. Statistical significance will be accepted at *p*-values of less than 0.05 (two-sided testing).

#### Secondary analysis

Similar analyses will be performed for the secondary time points T3 and T6, as well as for the total follow-up period, and with all secondary outcome measures. Only Global Perceived Effect (GPE) will be analyzed as a dichotomous variable (‘completely recovered’ and ‘much recovered’ vs. all other responses) using logistic multilevel analysis. Furthermore, effect sizes from all clinical outcome measures will be calculated.

#### Economic evaluation

For the economic evaluation, a trial-based cost-utility analysis will be performed, relating costs to quality-adjusted life years (QALYs). Societal costs will include healthcare utilization, informal care and work-related costs, assessed using patient questionnaires at 3, 6 and 12 months. QALYs will be calculated using the Dutch tariff for the EQ-5D-5L [65], assessed at baseline, 3, 6, and 12 months. Analyses will be performed in accordance with the Dutch guidelines for economic evaluations [66], with extrapolation beyond the one-year trial period. The primary analysis will be from a societal perspective with friction cost method to value productivity, whereas secondary analyses follow the human-capital approach and a healthcare perspective [67]. Costs will be related to outcome using net-benefit analysis, with multiple imputation to account for missing data.

#### Data management

All the data of the participants will be pseudonymized with assignment of a study number to every participant. The key to the study numbers will be stored in a separate file on the server of the Leiden University Medical Center (LUMC). Only the research team, an auditor from the LUMC and national and international supervisory authorities can access the participants’ personal information. The collected data will be stored for 15 years on a local server at the LUMC and a backup of the data will be stored at the LUMC.

## Discussion

Work ability of people with RA or axSpA is considerably reduced compared to the general population [8]. Despite the observed need for vocational interventions, research on the (cost-)effectiveness of vocational interventions is limited. To our knowledge, this is the first study on the (cost-)effectiveness of a PT-led, vocational intervention in people with RA or axSpA. Based on existing evidence and clinical experience, we have integrated all potentially effective treatment modalities into a single intervention, delivered by PTs specifically trained for this purpose. We expect that the incorporation of these individually effective modalities, with an embedded focus on work, will lead to a moderate effect as well as substantial cost savings through reduced sick leave and improved work productivity. Therefore, we hypothesize that a multimodal, PT-led, vocational intervention in (self-)employed people with RA or axSpA and a reduced work ability is effective and cost-effective compared to usual care.

We would like to acknowledge two (potential) study limitations about the population. First, as we include two patient groups (i.e., RA and axSpA), our study sample could be considered relatively heterogenous. However, people with RA as well as axSpA experience comparable symptoms, including joint pain, stiffness, fatigue [1–3] and reduced health-related quality of life [3, 4]. Furthermore, we include a homogenous sample from a work perspective, namely people with reduced work ability but still at work or less than 6 months on sick leave, as this population is expected to profit most from our intervention. Therefore, we expect these two patient groups to respond similarly to our intervention. Second, since participants in this study cannot be blinded to their randomization, it cannot be ruled out that control participants will seek a similar (PT) intervention that may lead to contamination and thereby reduce the contrast between our two arms. However, we do not expect this to occur, as work is usually not addressed in current practice [68, 69]. Nevertheless, we will carefully record the use of any healthcare or other services during the 12-months of follow-up. This enables us to demonstrate the level of contamination.

Furthermore, we would like to mention three (potential) limitations regarding the design of the study. First, the number of sessions in our intervention (10 to 21 sessions during a 12-month follow-up period) appears to be relatively extensive compared to other vocational intervention studies in IA, in which the number of sessions was between one and 12 [15–21]. Due to the complexity of the concept of work ability, a multimodal approach with a long follow-up period is considered necessary to achieve sustainable changes in work ability. Second, although we tested a draft version of our intervention for feasibility in a small group of four patients, unexpected barriers in its execution may arise if applied on a larger scale which could impact the outcomes of the study. Third, for practical reasons, we only include people in whom the cost of the intervention is covered by their complementary health insurance (or if not, people are willing to pay it out-of-pocket). This may lead to a selection bias, although a majority of the people with IA (namely 76% [25]) in the Netherlands have this coverage. Because we carefully capture this eligibility criterium (inclusion criterium 6), we have insight into the extent of this barrier after the completion of the trial.

To conclude, as the study is still ongoing the results are not available yet, the scientific implications of this publication are limited. However, given the fact that limited research on the effectiveness of interventions to increase the work ability of people with RA or axSpA is available, for those interested in the evidence on this topic it is important to be aware of ongoing studies. The results of this study will provide insights in the (cost-)effectiveness of a multimodal, PT-led, vocational intervention in people with RA and axSpA and a reduced work ability.

## Data Availability

The data generated during this study will not be publicly available, but will be available upon reasonable request to the corresponding author.

## Abbreviations

IA: Inflammatory arthritis
RA: Rheumatoid Arthritis
axSpA: Axial SpondyloArthritis
RCT: Randomized Controlled Trial
PT: Physiotherapist
OT: Occupational Therapist
WAS: Work Ability Index – Single Item Scale
PSC NRS: Patient Specific Complaints Numeric Rating Scale
EQ-5D-5L: EuroQol
GPE: Global Perceived Effect
QALY: Quality-adjusted Life Year
ICTRP: International Clinical Trial Registry Platform
SAE: Serious Adverse Event
AE: Adverse Event
LUMC: Leiden University Medical Center
